# Animal Model To Study Klebsiella pneumoniae Gastrointestinal Colonization and Host-to-Host Transmission

**DOI:** 10.1128/IAI.00071-20

**Published:** 2020-10-19

**Authors:** Taylor M. Young, Andrew S. Bray, Ravinder K. Nagpal, David L. Caudell, Hariom Yadav, M. Ammar Zafar

**Affiliations:** a Department of Microbiology and Immunology, Wake Forest School of Medicine, Winston-Salem, North Carolina, USA; b Department of Molecular Medicine, Wake Forest School of Medicine, Winston-Salem, North Carolina, USA; c Department of Pathology-Comparative Medicine, Wake Forest School of Medicine, Winston-Salem, North Carolina, USA; Stanford University

**Keywords:** *Klebsiella*, transmission, animal models, antibiotic resistance, capsular polysaccharide, enteric pathogens, fimbriae, hospital infections, host-pathogen interactions

## Abstract

An important yet poorly understood facet of the life cycle of a successful pathogen is host-to-host transmission. Hospital-acquired infections (HAI) resulting from the transmission of drug-resistant pathogens affect hundreds of millions of patients worldwide. Klebsiella pneumoniae, a Gram-negative bacterium, is notorious for causing HAI, with many of these infections difficult to treat, as K. pneumoniae has become multidrug resistant. Epidemiological studies suggest that K. pneumoniae host-to-host transmission requires close contact and generally occurs through the fecal-oral route. Here, we describe a murine model that can be utilized to study mucosal (oropharynx and gastrointestinal [GI]) colonization, shedding within feces, and transmission of K. pneumoniae through the fecal-oral route. Using an oral route of inoculation, and fecal shedding as a marker for GI colonization, we showed that K. pneumoniae can asymptomatically colonize the GI tract in immunocompetent mice and modifies the host GI microbiota. Colonization density within the GI tract and levels of shedding in the feces differed among the clinical isolates tested. A hypervirulent K. pneumoniae isolate was able to translocate from the GI tract and cause hepatic infection that mimicked the route of human infection. Expression of the capsule was required for colonization and, in turn, robust shedding. Furthermore, K. pneumoniae carrier mice were able to transmit to uninfected cohabitating mice. Lastly, treatment with antibiotics led to changes in the host microbiota and development of a transient supershedder phenotype, which enhanced transmission efficiency. Thus, this model can be used to determine the contribution of host and bacterial factors toward K. pneumoniae dissemination.

## Abstract (precis)

An important yet poorly understood facet of the life cycle of a successful pathogen is host-to-host transmission. Hospital-acquired infections (HAI) resulting from the transmission of drug-resistant pathogens affect hundreds of millions of patients worldwide. Klebsiella pneumoniae, a Gram-negative bacterium, is notorious for causing HAI, with many of these infections difficult to treat, as K. pneumoniae has become multidrug resistant. Epidemiological studies suggest that K. pneumoniae host-to-host transmission requires close contact and generally occurs through the fecal-oral route.

## INTRODUCTION

Host-to-host transmission of pathogens is the primary source of nosocomial infections, which are considered a serious threat to patients’ health and also a significant burden on the health care system (1, 2). Hospital-acquired infections (HAI) account for ∼100,000 deaths in the United States alone (3). A leading cause of these hospital-acquired infections and multiple outbreaks in hospitals around the world is Klebsiella pneumoniae, a member of the *Enterobacteriaceae* family that frequently causes pneumonia, bacteremia, pyogenic liver abscesses, and urinary tract infections (4), with most of these infections generally occurring in immunocompromised patients. With the rampant use of antibiotics, K. pneumoniae isolates have become extensively drug resistant, and some are now even considered pan-drug resistant, making the infections they cause extremely difficult to treat (5–7). For this reason, the WHO lists Klebsiella pneumoniae as a critical pathogen for which new antibiotics and other therapies are urgently required to address this growing health care problem (8, 9). Further exacerbating treatment of K. pneumoniae infections is the recent identification of isolates termed hypervirulent K. pneumoniae (hvKP) that can cause disease, such as community-acquired pyogenic liver abscesses in healthy individuals (10–12). Patients recovering from hvKP infections often suffer from postinfectious sequelae that can lead to loss of limb or vision (13–15). These strains, originally isolated in the Pacific Rim, have since disseminated worldwide (10).

In the natural environment, the initial mucosal sites of colonization tend to be the oropharynx and the gastrointestinal (GI) tract (16, 17). These colonization events are generally asymptomatic (18). However, under certain circumstances, K. pneumoniae can gain access to other sterile sites in the host and cause disease. Epidemiological data suggest that many patients in hospitals carry K. pneumoniae in the GI tract, with a correlation between K. pneumoniae carriage and subsequent disease from the same isolate (19–21). Besides patients, hospital personnel can also be asymptomatic carriers of K. pneumoniae, and these silent carriers act as a reservoir from which K. pneumoniae can manifest disease within the same host or act as a source of transmission to a new host (18, 22–24).

Colonization resistance provided by the host microbiota plays a critical role in blocking colonization by pathogens. However, the use of antibiotics diminishes the microbial diversity in the GI tract, which potentially allows K. pneumoniae to readily colonize a host. Studies also show that antibiotic treatment of mice predisposes them to a “supershedder” state in which they shed resident gut pathogens at a higher number, which enhances host-to-host transmission (25, 26). It is, however, unclear whether antibiotic treatment in a hospital setting contributes toward the increased transmission of drug-resistant K. pneumoniae.

Our understanding of the Klebsiella pneumoniae-associated disease state comes mainly from animal models studying lung and urinary tract infections. While these studies have identified bacterial and host factors that contribute to K. pneumoniae virulence, there is very little mechanistic understanding of the gastrointestinal colonization and host-to-host transmission. Close contact, especially in a hospital setting, is thought to promote the spread of K. pneumoniae from an infected host to a naive host. Transmission is thought to occur via the fecal-oral route, through either poor hygiene or contact with contaminated surfaces (fomites) (20, 22–24).

Here, we describe a murine model to allow for the study of K. pneumoniae GI colonization, shedding, and host-to-host transmission. Employing an oral route of K. pneumoniae inoculation in an inbred mouse population, we investigated K. pneumoniae gastric colonization and transmission. We demonstrate that K. pneumoniae can stably colonize the GI tract without treatment with antibiotics, and that these mice stay persistently colonized and can transmit K. pneumoniae to cagemates. Furthermore, antibiotic treatment of carrier mice induces gut dysbiosis and triggers a transient supershedder phenotype.

## RESULTS

### Establishing Klebsiella pneumoniae colonization in the murine intestinal tract.

**FIG 1:**
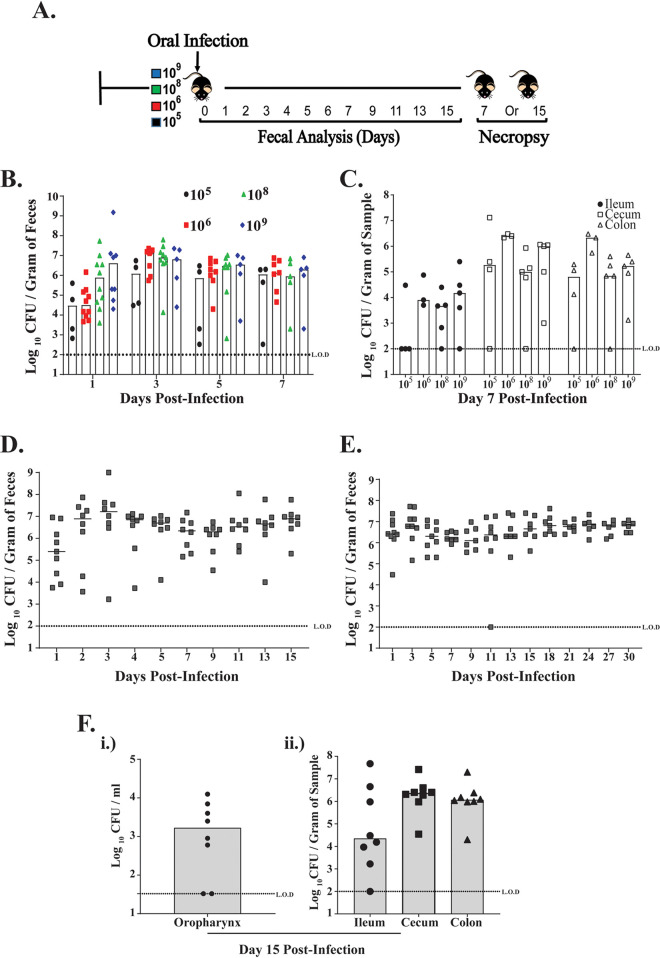
K. pneumoniae colonizes the gastrointestinal tract of mice. (A) A schematic representation of C57BL/6J mice orally infected with K. pneumoniae KPPR1S isolate (10^5^, 10^6^, 10^8^, and 10^9^ CFU) (*n* ≥ 4 mice in each group). (B and C) Quantified daily shedding results and colonization density in the intestinal tract (ileum, cecum, and colon) at day 7 postinoculation from mice given different K. pneumoniae doses. (D and E) Fecal shedding data collected from mice given 10^6^ CFU of KPPR1S and followed for up to either 15 days or 30 days postinfection (*n* = 9 mice in each group). (F) (i and ii) Colonization density of KPPR1S isolate in the oropharynx and lower GI tract of mice 15 days postinoculation. Bars indicate the median values. L.O.D, limit of detection.

We sought to establish a GI model of Klebsiella pneumoniae colonization that would mimic natural colonization in a host. Because of the difficulty in establishing K. pneumoniae GI colonization through gavage treatment, previous studies used antibiotic pretreatment to disrupt the host microbiota and allow for K. pneumoniae colonization via the gavage method (27–29). We first tested the ability of K. pneumoniae to colonize the GI tract by giving adult mice doses ranging from 10^5^ to 10^9^ CFU/100 μl, without antibiotic treatment so as not to disrupt the host microbiota (Fig. 1A). However, instead of a gavage treatment, mice were infected orally by pipette feeding to simulate the natural route of infection (30). We used K. pneumoniae clinical isolate KPPR1S, which has been used extensively to model the K. pneumoniae-associated disease state in mice. The streptomycin and rifampin resistance of KPPR1S allowed us to enumerate the bacteria in the fecal pellets on selective plates. As shown in Fig. 1B and C, K. pneumoniae colonized the GI tract and was shed robustly in the feces of mice with doses above 10^5^ CFU. A dose of <10^5^ CFU did not result in the establishment of colonization (threshold for detection, 100 CFU), suggesting that a minimum dose of 10^5^ is required (data not shown). Based upon these results, we chose 10^6^ CFU as the minimum dose required to establish K. pneumoniae colonization. Based on our preliminary studies that suggest that poor K. pneumoniae fecal shedding correlates with reduced GI colonization, we used daily fecal shedding as a substitute for colonization density in the GI tract. Next, we determined how long K. pneumoniae colonizes the mouse GI tract. We followed K. pneumoniae shedding in feces of infected mice for either 15 or 30 days postinoculation (p.i.) and observed that K. pneumoniae was shed at similar levels throughout the study (Fig. 1D and E). Furthermore, our results showed that K. pneumoniae colonizes the mucosal surface of the oropharynx (Fig. 1F). Taken together, our data suggest that when introduced by the oral route, K. pneumoniae colonizes the mucosal surface of the oropharynx, can establish and persist in the GI tract, and is shed robustly in the feces.

**FIG 2:**
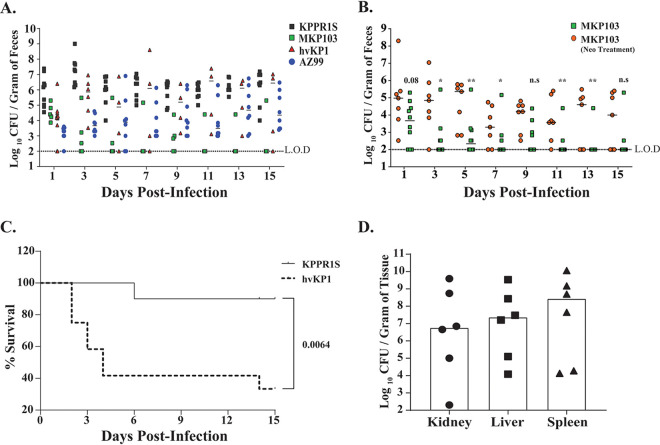
Differences among K. pneumoniae clinical isolates in fecal shedding levels and virulence. (A) Mice were infected with the indicated K. pneumoniae isolate, with daily shedding values shown (*n* ≥ 5 mice for each isolate). Differences in total shedding over the course of infection (15 days) between different K. pneumoniae isolates were determined using the Kruskal-Wallis test. *P* < 0.001 (KPPR1S versus MKP103), not significant (KPPR1S versus hvKP1), *P* < 0.05 (KPPR1S versus AZ99). (B) Comparison between mice infected with K. pneumoniae strain MKP103 with or without pretreatment of antibiotic neomycin (Neo; 5 mg/200 μl) (*n* ≥ 7 mice in each group). Symbols represent shedding values obtained from a single mouse on a given day. The bar represents the median value. The Mann-Whitney test was used to determine the differences in fecal shedding. (C) Survival of mice infected gastrointestinally with K. pneumoniae isolate KPPR1S (*n* = 10) or hvKP1 (*n* = 10) over 15 days. An in-extremis state or death was scored as nonsurvival. Log-rank (Mantel-Cox) test was performed to determine statistical differences in survival. (D) Bar graph showing median colonization density of hypervirulent isolate hvKP1 in the kidney, liver, and spleen from mice that were initially infected orally. Each data point represents colonization density in a specific organ from a specific mouse. Organs were harvested from mice that displayed an in-extremis state. The limits of detection were 33 CFU/ml for liver and kidney and 10^2^ CFU/ml for spleen. *, *P* < 0.05; **, *P* < 0.01; ***, *P* < 0.001.

**FIG 3:**
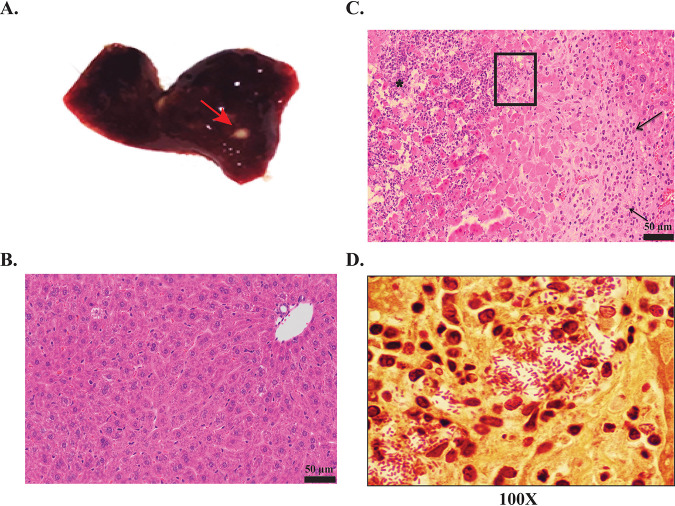
(A) Representative K. pneumoniae liver abscesses (arrow) from mice that succumbed to infection from the hvKP1 isolate that originally colonized the GI tract. (B and C) Liver tissue samples stained with hematoxylin and eosin staining. Shown are liver tissue samples at 20× resolution from either uninfected mice showing normal hepatocytes (B) and infected mice that contains a large region of coagulative and lytic necrosis with hepatocellular disassociation including central infiltrates of neutrophils (*) with few scattered mononuclear inflammatory cells (C). The peripheral aspect of the lesion contains numerous lymphocytes, macrophages, and new scattered neutrophils (arrows). (D) 100× oil immersion image of the boxed area from panel C showing Gram-negative bacteria present within the necrotic liver tissue.

A hallmark of K. pneumoniae isolates is their genetic heterogeneity, which affects their ability to cause disease (31). Thus, we determined whether K. pneumoniae genetic plasticity also contributes to GI colonization. We tested the ability of a set of genetically diverse clinical K. pneumoniae isolates to colonize the GI tract in mice. For analysis we chose MKP103, a derivative of KPNIH1, which was the cause of an outbreak at NIH Clinical Center, hvKP1, a hypervirulent human isolate, and AZ99, a human fecal isolate. All three strains showed various densities of colonization of the murine GI tract, with hvKP1 shedding at a level similar to that of KPPR1S (Fig. 2A). Surprisingly, the MKP103 isolate colonized poorly, with mice generally clearing it from the GI tract by day 5 p.i. As observed through fecal shedding, the human fecal isolate AZ99 consistently colonized the GI tract, albeit at a lower density than KPPR1S. Moreover, mice colonized with hvKP1 had a high mortality rate (Fig. 2C). Hypervirulent isolates are notorious for causing pyogenic liver abscesses (PLA) (11). As shown in Fig. 2D, mice that succumbed after oral inoculation with the hvKP1 isolate were colonized at a high density in the liver, kidney, and spleen with the same isolate. Moreover, these mice appeared to have developed liver abscesses (Fig. 3A), which hematoxylin and eosin (H&E) and Gram staining confirmed to contain necrotic tissue, inflammatory cells, and Gram-negative bacteria (Fig. 3B to D). Development of liver abscess was a common outcome for mice infected with the hvKP1 isolate. Thus, our model mimics human disease dynamics, in which a hypervirulent isolate (hvKP1) is able to translocate from the GI tract to other sterile sites and cause the development of the disease state.

### Antibiotic treatment leads to the development of the K. pneumoniae supershedder phenotype.

Given that the fecal-oral route of transmission in a hospital setting is considered a significant cause of nosocomial infections (32, 33), it was surprising that the MKP103 isolate failed to colonize the GI tract in mice (Fig. 2A). However, as many of the patients that acquired MKP103 in the GI tract were on antibiotics (20), we considered whether the use of antibiotics would affect the ability of this isolate to colonize the GI tract. Moreover, high use of antibiotics in a health care setting correlates with Klebsiella pneumoniae infections (20). Therefore, mice were treated with neomycin by gavage to reduce the colonization resistance by the host GI microbiota and then infected with MKP103 to determine whether antibiotic treatment positively affected its ability to colonize. As shown in Fig. 2B, antibiotic pretreatment of mice allowed MKP103 to colonize and persist within the infected host GI tract up to 15 days postinfection.

**FIG 4:**
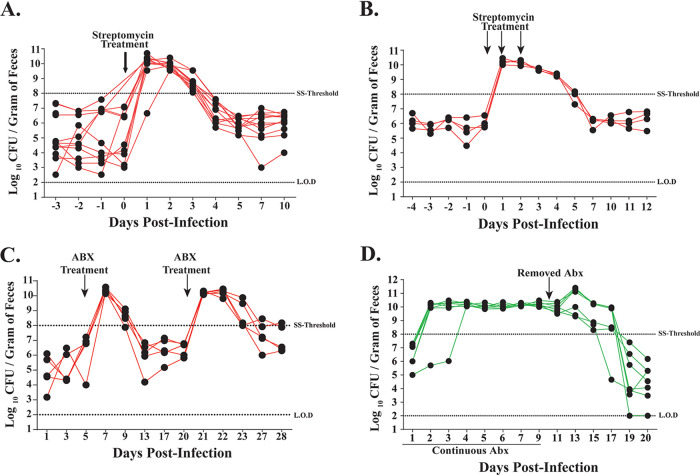
Antibiotic treatment of K. pneumoniae-infected mice triggers the supershedder phenotype. Fecal shedding from individual mice (*n* = 9) infected with K. pneumoniae KPPR1S given a single dose of streptomycin sulfate (5 mg/200 μl) (A) or (*n* = 4) given three treatments of streptomycin on consecutive days (B) resulted in a rapid development of high shedding (>10^8^ CFU/gram of feces, i.e. supershedder [SS] threshold) lasting for 3 days post antibiotic treatment. (C) Streptomycin sulfate (5 mg/200 μl) treatment triggered the supershedder phenotype (>10^8^ CFU/gram of feces) in mice (*n* = 4) infected with K. pneumoniae. A second treatment with streptomycin sulfate (5 mg/200 μl) once mice returned to basal K. pneumoniae shedding elicited a second transient supershedder state. (D) High fecal shedding of K. pneumoniae isolate MKP103 from mice (*n* = 6) given ampicillin (1 g/liter) in drinking water. Removal of antibiotic pressure began an eventual shift to reduced fecal shedding (<10^8^ CFU/gram of feces).

Our results show that antibiotic treatment allows a K. pneumoniae isolate (MKP103) that colonizes poorly to establish itself in the GI tract. However, whether antibiotic treatment affects colonization density of isolates that colonize robustly without requiring antibiotic treatment remains unknown. We determined whether treatment with antibiotics would lead to the development of a supershedder phenotype, in which an infected host sheds the pathogen at a much higher number than another infected host. This phenomenon has been observed in the natural setting and is considered a major source of host-to-host transmission (34). Murine models have been used to characterize this phenotype, for which >10^8^ CFU/g (supershedder [SS] threshold) of the indicated pathogen in the feces is generally considered the threshold for the supershedder phenotype (SS phenotype) (25, 26). Using the KPPR1S isolate, as it consistently colonized mice at a high density without antibiotic treatment, we assessed fecal shedding of K. pneumoniae for 10 to 12 days p.i. after either a single streptomycin treatment or three consecutive days of streptomycin treatment. We found that antibiotic treatment triggered a temporary supershedder phenotype (Fig. 4A and B), whereas no such phenotype was observed with the vehicle-only control (phosphate-buffered saline [PBS]) (see Fig. S1 in the supplemental material). A second treatment of antibiotics, after mice had returned to baseline levels of K. pneumoniae shedding from the first antibiotic treatment, caused the development of another transient supershedder phenotype (Fig. 4C).

In a clinical setting, immunocompromised patients tend to be on continuous antibiotic treatment; therefore, we determined the effect of daily antibiotic treatment on K. pneumoniae shedding. We supplemented the drinking water of mice with ampicillin 24 h before K. pneumoniae inoculation and continued for 10 days p.i. As K. pneumoniae is intrinsically resistant to ampicillin, the mice infected with MKP103 isolate displayed the K. pneumoniae supershedder phenotype (Fig. 4D). After removal of antibiotic pressure, the mice displayed the high-shedding phenotype for multiple days. Taken together, our data suggest that as a consequence of antibiotic treatment, K. pneumoniae can develop a supershedder phenotype and the length of this phenotype is dependent upon the duration of the antibiotic treatment.

### Antibiotic treatment leads to the disruption of host microbiota that correlates with the supershedder phenotype.

**FIG 5:**
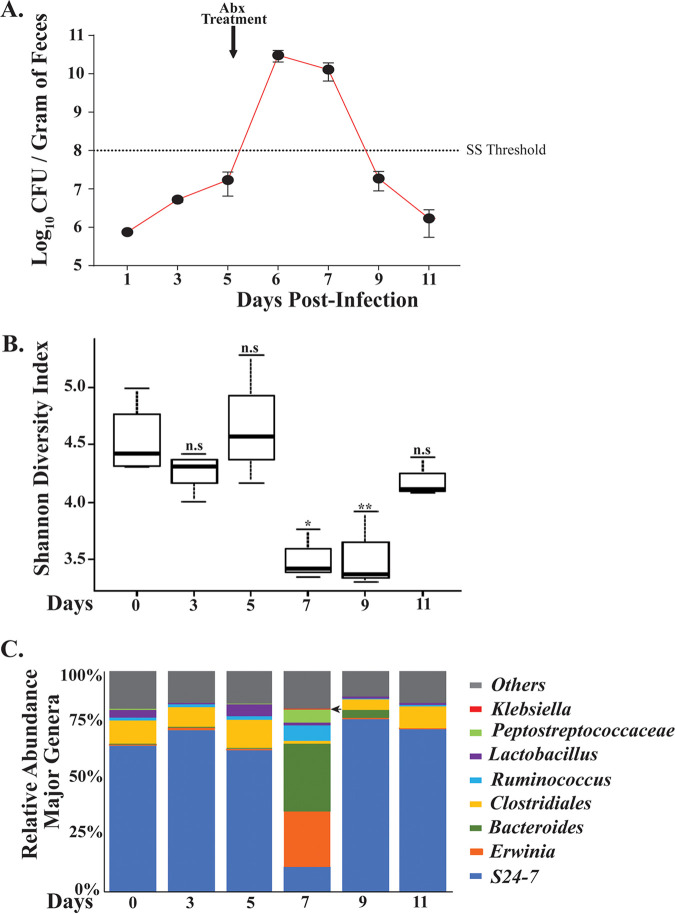
Antibiotic-triggered supershedder phenotype correlates with reduced intestinal microbial diversity. (A) Average fecal shedding of K. pneumoniae isolate KPPR1S by carrier mice (*n* = 4) pre-antibiotic treatment and post-single-dose-streptomycin treatment (5 mg/200 μl) that induces a transient supershedder phenotype. Error bars represent SEMs. (B) Intestinal microbiota changes in carrier mice from samples obtained on day 0 preinfection, days 3 and 5 postinoculation, and days 7, 9, and 11 post-antibiotic treatment. DNA was isolated from fecal samples obtained from infected mice and 16S rRNA analysis was carried out. Data are shown as Shannon diversity index, with means and SDs shown. Statistical differences were calculated by Kruskal-Wallis test. (C) Shift in microbial diversity determined from fecal samples collected on the days indicated and shown as bar graph with percent relative abundance of major genera. Arrow shows identification of Klebsiella pneumoniae DNA at day 7 postinfection. *, *P *< 0.05; **, *P* < 0.01.

Next, we determined whether the K. pneumoniae infection- or antibiotic treatment-induced supershedder phenotype is a result of the displacement of the host microbiota. To provide insight into the K. pneumoniae carrier state and the supershedder phenotype, we carried out a 16S rRNA analysis to determine the host intestinal microbiota changes that occurred during infection and as a consequence of antibiotic treatment (Fig. 5A). For a detailed 16S rRNA analysis, we isolated DNA from fecal samples collected at six different time points from K. pneumoniae-infected mice (*n* = 4). Fecal pellets were collected preinoculation to determine the baseline of the host GI microbiota. Samples were collected on days 7, 9, and 11 post-antibiotic treatment to determine changes in the host microbiota. At days 3 and 5 p.i., we were unable to detect K. pneumoniae 16S rRNA gene sequences, even though K. pneumoniae was shed at 10^6^ CFU/g of fecal sample. This result suggests that K. pneumoniae comprises only a minor component of the host intestinal microbiota. The main component of a diverse microbial community of the host intestine included *Bacteroidetes* (*Bacteroidales* [S24-7]) and *Firmicutes* (*Clostridiales*) (Fig. 5C; Table S1), which are considered to constitute a typical profile for stable mammalian intestinal microbiota.

A single treatment with streptomycin (Str) led to dramatic changes in the intestinal microbiota. As detailed in Fig. 5B, there was a statistically significant decline in the total species richness, especially in S24-7, with a concurrent increase in *Erwinia* and *Bacteroides*. As illustrated in Fig. 5C, we observed K. pneumoniae-specific 16S rRNA gene sequences only for the antibiotic-induced supershedder phenotype. A decrease in K. pneumoniae shedding levels correlated with an increase in S24-7 and other major components of the host microbiota and a loss of detection of K. pneumoniae-specific 16S rRNA sequences. Thus, antibiotic treatment leads to a disruption of host microbiota that correlates with the development of a temporary supershedder phenotype. Moreover, disruption of the host microbiota with antibiotics is associated with reduced microbial richness, which recovers 3 days post-antibiotic exposure.

### Klebsiella pneumoniae factors contributing to shedding and colonization.

**FIG 6:**
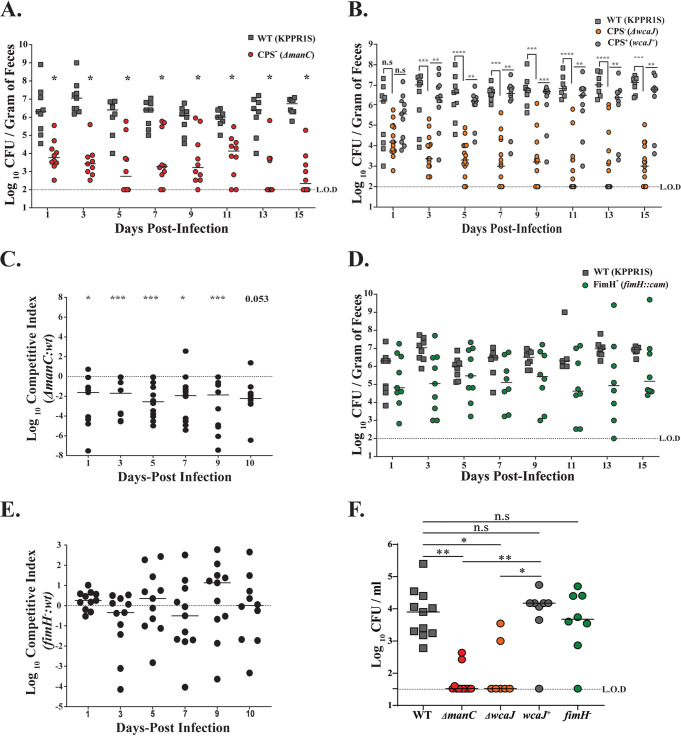
Effects of K. pneumoniae virulence factors on fecal shedding and oropharyngeal colonization. (A and B) Mice were infected orally with WT KPPR1S, one of the capsule-deficient mutants (*ΔmanC* and *ΔwcaJ*), or the chromosomally complemented strain (*wcaJ^+^*), and feces were collected on the days indicated (*n* ≥ 10 for each group). Each symbol represents CFU obtained from a single mouse on a given day, with solid lines representing median values. Mann-Whitney test was used to determine the differences in fecal shedding between the WT and *ΔmanC* strains. Kruskal-Wallis test was used for comparing the WT with *ΔwcaJ* and *wcaJ*^+^ strains. No statistical differences were observed in shedding between the WT and *wcaJ*^+^ strains. (C) Mice were infected orally with a 1:1 mixture of the WT and the capsule-deficient mutant (*ΔmanC*), with feces collected on the days indicated (*n* ≥ 10). The CI was determined as described in Materials and Methods. Each symbol represents the log_10_ CI value from an individual mouse on a given day. The solid line represents the median. The dashed line indicates a competitive index of 1 or a 1:1 ratio of mutant to WT. (D) Mice were infected orally with the WT or with an isogenic mutant (*fimH*::*cam*; FimH^−^), and feces were collected on the days indicated (*n* ≥ 10 for each group). (E) Mice were infected orally with a 1:1 mixture of the WT and the mutant (FimH^−^), and feces were collected on the days indicated. The CI was determined as mentioned in Materials and Methods (*n* ≥ 10). (F) The colonization density in the oropharynx for the WT isolate, the isogenic mutants, and the complemented strain was determined 15 days postinfection, with median values shown. For CI, statistical differences were determined by Wilcoxon signed-rank test. Differences in oropharyngeal colonization were determined using Kruskal-Wallis test. *, *P < *0.05; **, *P < *0.01; ***, *P < *0.001; ****, *P < *0.0001.

To examine the contributions of known virulence determinants of K. pneumoniae, we tested shedding and colonization of the previously described capsule (CPS)-deficient mutant (*ΔmanC*) of the strain KPPR1S. As is evident from Fig. 6A, over the course of 15 days of infection, the *ΔmanC* mutant shed and also colonized poorly (Fig. S2) in comparison to the parental wild-type (WT) strain. To provide further evidence of the role of CPS in GI colonization, we also tested a mutant with a deletion of *wcaJ* (35), which codes for a glycosyltransferase involved in initial steps of CPS production. Similar to the *ΔmanC* mutant, the *ΔwcaJ* mutant over the course of the 15 days shed poorly (Fig. 6B). Furthermore, when the *ΔwcaJ* mutant was chromosomally complemented (*wcaJ^+^*), it behaved as the parental WT strain (Fig. 6B; Fig. S2), suggesting that the colonization defect is specific to CPS.

Bacteria can form biofilm-like structures in the GI tract (36). We hypothesized that a coinfection with WT K. pneumoniae and the Δ*manC* strain would form a mixed population (intraspecies) biofilm in the GI tract, helping compensate for the capsule deficiency of the Δ*manC* strain. However, coinfected mice still shed the Δ*manC* strain poorly compared to the parental strain (Fig. 6C). These observations suggest that capsular polysaccharide of K. pneumoniae is essential for robust GI colonization and eventual fecal shedding.

Next, as the type 1 fimbria of K. pneumoniae is considered essential for colonization of the host urinary tract, we determined its role in GI colonization (37). The KPPR1S *fim* locus promoter is under phase-variable control, which was observed to be in the off position both *in vitro* (broth culture) and *in vivo* (fecal pellets) (data not shown). To determine the requirement of the type 1 fimbria of KPPR1S in GI colonization, a deletion mutant of *fimH*, which encodes the type 1 fimbria tip adhesin, required for proper interaction with the host epithelial layer (38), was constructed. As is evident from Fig. 6D and E, even though mice infected with the Δ*fimH* mutant had reduced median shedding, shedding was not significantly lower than for the WT strain. Lastly, we determined whether these mutants also contribute toward colonization of the mucosal surface of the oropharynx. Figure 6F shows that capsule was essential for colonization of the oropharyngeal space, whereas the type 1 fimbria was dispensable. Overall, our data indicate that K. pneumoniae capsular polysaccharide plays a critical role in GI colonization. In contrast, the K. pneumoniae type 1 fimbria appears to be nonessential for gut colonization.

### Klebsiella pneumoniae transmission occurs through the fecal-oral route.

**FIG 7:**
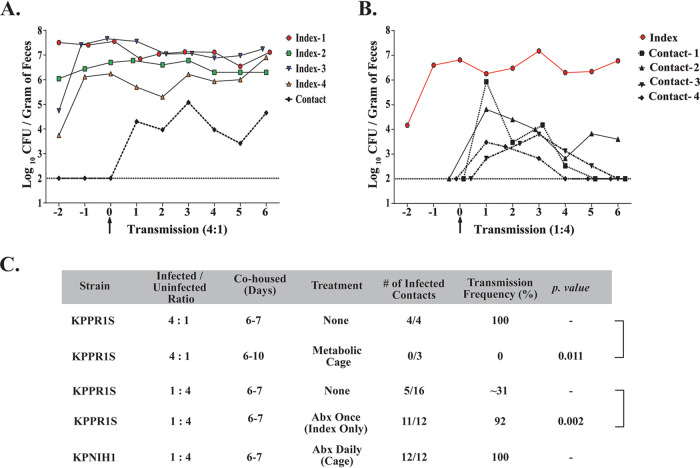
K. pneumoniae transmission between hosts with and without treatment of antibiotics. (A) A representative of 4:1 ratio of index-to-contact transmission. Four naive mice were infected orally with KPPR1S and housed with one uninfected mouse. Fecal shedding was monitored daily from both the index mouse and the contact mouse. (B) A representative of 1:4 ratio of index-to-contact transmission. One naive mouse was infected orally with KPPR1S and housed with four uninfected mice, and fecal shedding was monitored daily (C) Observed efficiency of K. pneumoniae transmission with different ratios of infected to uninfected mice, effect of antibiotics, and transmission dynamics in a metabolic cage. Statistical differences calculated using two-tailed Fisher’s exact test. In all transmission studies, fecal colonization was observed within 24 h. In 1:4 no-treatment transmission studies (*n* = 4), at least one successful transmission event was observed.

Transmission of enteric pathogens generally occurs through the fecal-oral route, and host-to-host transmission in a hospital setting is a major source of infection (20, 32). Thus, we determined whether K. pneumoniae host-to-host transmission events could be observed in our animal model. Initially, we housed one uninfected mouse (contact) with four infected mice (index). Fecal pellets were collected to enumerate colonization density and whether transmission from index to contact mice occurred. We observed 100% transmission efficiency with a ratio of 4:1, with transmission occurring within 24 h of cohousing the animals in all of our experiments (Fig. 7A and C). Since transmission efficiency is high with a ratio of 4:1, we decided to determine K. pneumoniae transmission dynamics with one index mouse cohoused with four contact mice. With a ratio of 1:4, reduced transmission efficiency (∼31%) was observed, suggesting that not enough K. pneumoniae shedding events occurred for all uninfected mice to become colonized (Fig. 7B and C). However, in all of the 1:4 ratio transmission studies (*n* = 4), we observed at least one successful transmission event in each of the experiments.

Next, to mimic conditions prevalent in a hospital, where patients tend to be on antibiotics, we investigated the effects of antibiotic treatment on K. pneumoniae transmission dynamics. A single antibiotic treatment to the index mouse cohoused with four contact mouse led to >90% transmission, suggesting that high K. pneumoniae shedding in the fecal pellets can overcome colonization resistance of the contact mice (Fig. 7C). Lastly, we tested the effect of antibiotics on both index and contact mice by adding antibiotics in their drinking water. An index mouse was infected with MKP103 and was housed separately for several days before being introduced to four contact mice already on antibiotics. We observed 100% transmission efficiency when both index and contact mice were on daily antibiotics. Moreover, as all the mice in the cage were on antibiotics, they all developed the supershedder phenotype (Fig. S3A). Our results provide insight into the high transmissibility of K. pneumoniae in hospitals where there is high antibiotic usage.

We hypothesize that K. pneumoniae transmission occurs through the fecal-oral route, based upon transmission models for other enteric pathogens and the coprophagic nature of mice. However, as K. pneumoniae colonizes both the oral cavity and the GI tract, we sought to determine whether host-to-host transmission of K. pneumoniae is due to the coprophagic action of mice or occurs by contact with infected oral secretions. Mice were housed in a metabolic cage, where they did not have access to their fecal pellets. At a 4:1 ratio of the index to contact mice, no transmission events were detected between the infected and the uninfected mice during the 10-day experiment, suggesting that in our animal model, host-to-host transmission requires contact with fecal matter (Fig. 7C). Lastly, the infected mice in the metabolic cage shed K. pneumoniae robustly compared to infected mice housed in regular cages, suggesting persistent colonization did not require reseeding through the consumption of infected fecal pellets (Fig. S3B).

## DISCUSSION

The genetic heterogeneity of Klebsiella pneumoniae allows this pathogen to colonize a variety of host mucosal surfaces, which can dramatically impact the clinical outcome. Klebsiella pneumoniae disease manifestations in the respiratory and urinary tract have been extensively modeled in animals (4). However, the gastrointestinal mucosal surface, also colonized readily by K. pneumoniae, has not been the focus of many scientific studies (39, 40). In this report, we describe a murine model of oral infection of K. pneumoniae to study GI colonization and host-to-host transmission. We demonstrate for the first time that K. pneumoniae can stably colonize the GI tract of immunocompetent mice without disrupting the host microbiota, a key strength of our model. Second, a host colonized persistently with a pathogen is considered a significant reservoir for new infections, and our animal model of K. pneumoniae GI colonization replicates this phenotype, indicating it is a useful tool to study within-host events and host-to-host transmission. Third, we observed variability in the ability to colonize the GI tract and to cause invasive disease between different K. pneumoniae isolates, suggesting that K. pneumoniae genetic plasticity might be involved in the observed variability. Lastly, as many patients in a hospital setting tend to be on antibiotics, we were able to show experimentally for the first time that antibiotic treatment triggers the development of the supershedder phenotype in carrier mice, which promotes host-to-host transmission.

Previous studies used antibiotic treatment to reduce host colonization resistance by disrupting the resident microbiota to establish K. pneumoniae colonization (27, 37, 41). However, treatment with antibiotics reduces the ability to discern the role of bacterial factors that allow K. pneumoniae to overcome colonization resistance. Our model does not require the use of antibiotics to establish stable and persistent K. pneumoniae colonization, and therefore, it allows for the identification of bacterial and host factors that contribute to K. pneumoniae colonization and transmission. In our initial studies, we established that the route of infection is critical for stable colonization of K. pneumoniae in the GI tract. Oral gavage, a standard mode of infection for modeling enteric infections in murine models, only led to transient K. pneumoniae colonization in the GI tract (Fig. S4). However, orally feeding a similar dose allowed K. pneumoniae to colonize the GI tract and persist without disrupting the host microbiota. A recent study by Atarashi et al. showed that K. pneumoniae colonizing the oral cavity of patients can seed the GI tract (30). In our model of infection, we also observed K. pneumoniae colonizing the murine oral cavity. How oral feeding promotes robust K. pneumoniae colonization compared to the gavage method remains to be determined. It is possible that the oral route provides a mechanism though which K. pneumoniae is primed to colonize the GI tract. Future studies will aim to provide an understanding of K. pneumoniae priming that promotes its colonization of the GI tract.

A hallmark of many GI pathogens is their ability to cause an acute host inflammatory response. Multiple reports also suggest that K. pneumoniae might contribute toward gut dysbiosis and play an active role in inducing host response (30, 42). However, epidemiological data also suggest that K. pneumoniae can silently colonize healthy individuals (18). In our murine model, we were unable to detect any acute signs of inflammation post-K. pneumoniae infection. Furthermore, unlike the Salmonella enterica serovar Typhimurium supershedder phenotype, the K. pneumoniae antibiotic-induced supershedder phenotype was not associated with colitis (Fig. S5). Our data suggest that K. pneumoniae (KPPR1S isolate) in the GI tract behaves in a manner that generally does not elicit an acute inflammatory response and carriage is considered an asymptomatic event.

The roles of major virulence factors of K. pneumoniae, including its capsular polysaccharide (CPS), type 1 fimbriae, and others, have been extensively examined under both *in vitro* and *in vivo* conditions (4). However, data for the requirement of K. pneumoniae CPS in GI colonization appear to be contradictory (27, 28, 43, 44). As those studies were undertaken with mice treated with antibiotics, it is possible that the exact role of the bacterial factors was probably masked. Here, using our model, we show definitively that CPS of K. pneumoniae is an essential component required for efficient colonization of both the upper (oropharynx) and lower (intestinal) GI tract. The role of the capsule possibly pertains to protection against host-mediated clearance and interactions with mucus (28, 45, 46).

We also tested the requirement of the K. pneumoniae type 1 fimbria in GI colonization. Even though the type 1 fimbria is critical for colonization of the urinary tract, it appears to be dispensable for GI colonization (37). However, with certain pathogenic Escherichia coli isolates, the type 1 fimbria is required for colonization (47, 48). Furthermore, recent work by Jung et al. using antibiotic-treated mice observed a defect in GI colonization with a K. pneumoniae *fimD* mutant that is missing the usher constituent that facilitates assembly and eventual translocation of the pilus across the outer membrane (41). However, in our model we observed only a slight reduction in median shedding from mice infected with the *fimH* isogenic mutant compared to the WT strain, suggesting that the expression of the type 1 fimbria of the KPPR1S isolate is dispensable for GI colonization. This result showing that the type 1 fimbria of KPPR1S isolate does not contribute toward GI colonization was not unexpected, as we did not observe its expression under the conditions tested (data not shown). However, the type 1 fimbria might play a role in GI colonization for isolates that do express the structure.

Our model also allows us, for the first time, to understand the transmission dynamics of Klebsiella pneumoniae. We observed transmission between K. pneumoniae-infected and contact mice, suggesting that K. pneumoniae shedding in this model is high enough to permit transmission, albeit at a lower frequency. We also observed that the contact mice were colonized at a lower density than the index mice, possibly because of the reduced K. pneumoniae dose in the fecal pellets. Thus, our data follow epidemiologic studies that suggest 5 to 25% carriage rates in the natural environment (19, 49). However, the treatment of either infected or contact mice with antibiotics led to a high host-to-host transmission frequency. A single dose of antibiotic treatment established a supershedder phenotype in the index host, which was able to transmit to >90% of uninfected mice. It is suggested that 80% of the infections are due to 20% of infected individuals transmitting to uninfected hosts (known as the 80/20 rule) (50). Such individuals are termed as supershedders or superspreaders. However, we were unable to observe a K. pneumoniae supershedder phenotype without disrupting the host microbiota, suggesting that in our animal model, K. pneumoniae transmission dynamics do not follow the 80//20 rule. Multiple studies on enteric pathogens show that antibiotic treatment causes a dysbiosis in the GI tract, reduces colonization resistance by the stable resident microbiota, and promotes the expansion of pathogens (25, 26). Our 16S rRNA analysis shows that the antibiotic-based supershedder phenotype correlates with a reduction in microbial diversity. In contrast to *Salmonella* serovar Typhimurium and Clostridium difficile supershedder phenotypes (25, 26), the K. pneumoniae supershedder phenotype lasts for a shorter duration following a single antibiotic treatment. However, K. pneumoniae-infected mice on continuous antibiotics shed at supershedder levels, a condition we believe to be common in a hospital setting. Our data suggest that the development of the supershedder phenotype is the main contributor to host-to-host transmission events in a hospital. The transmission frequency of K. pneumoniae has not been established in a hospital setting and may be higher or lower than the rates determined in our murine model. We believe that a setting with high antibiotic use increases the likelihood of K. pneumoniae outbreaks. Therefore, patients on antibiotics should be carefully monitored to determine if they are colonized with K. pneumoniae.

In conclusion, we have described a model that will be useful in understanding complex interactions between K. pneumoniae and the host immune system and the intestinal microbiota. The availability of an arrayed marked mutant library of K. pneumoniae (51) and several annotated K. pneumoniae genomes should allow for studies identifying bacterial factors that contribute toward K. pneumoniae colonization and transmission. Since a majority of K. pneumoniae nosocomial infections arise from GI colonization and the fecal-oral route of transmission (20, 52), an understanding of the biology of K. pneumoniae gastrointestinal colonization and fecal-oral transmission would be valuable, as it could serve as an ideal point of intervention.

## MATERIALS AND METHODS

### Ethics statement.

This study was conducted according to the guidelines outlined by National Science Foundation animal welfare requirements and the *Public Health Service Policy on Humane Care and Use of Laboratory Animals* (53). The Wake Forest Baptist Medical Center IACUC oversees the welfare, well-being, and proper care and use of all vertebrate animals. The approved protocol number for this project is A18-160.

### Bacterial growth conditions and strain construction.

Strains used in the study are listed in Table 1. K. pneumoniae isolates were grown in Luria-Bertani (LB) (Lennox) broth, with constant agitation at 37°C. For all mouse infections, an overnight culture of K. pneumoniae was spun down at ∼27,000 × *g* for 15 min, and the resulting pellet was resuspended in a similar volume of 1× phosphate-buffered saline (PBS). To obtain the desired density for mouse infections (10^6^ CFU/100 μl), the bacterial suspension in PBS was diluted into a 2% sucrose-PBS solution. Ten-fold serial dilutions were plated on selective media (LB agar with antibiotic) and incubated at 30°C overnight for quantitative culture. LB plates contained antibiotics to select for specific K. pneumoniae isolates. Antibiotics used for isolating various isolates included streptomycin (Str; 500 μg/ml), chloramphenicol (50 μg/ml), ampicillin (25 μg/ml), apramycin (50 μg/ml), spectinomycin (30 μg/ml), and rifampin (30 μg/ml). Table 1 lists the resistance markers associated with the isolates used in this study.

**TABLE 1:** Strains used in this study

Strain	Description	Antibiotic resistance phenotype^*a*^	Reference or source
AZ10	K. pneumoniae stool isolate; ST 1322; *wzi* 372	Amp^r^	52
AZ99	Mouse-passaged Str^r^ derivative of AZ10	Amp^r^ Str^r^	This study
AZ17	KPPR1 serotype 2 K. pneumoniae isolate; derivative of ATCC 43816	Rif^r^	68
AZ55	KPPR1S; Str^r^ derivative of KPPR1	Rif^r^ Str^r^	69
AZ56	*ΔmanC* (CPS^−^) mutant of KPPR1S	Rif^r^ Str^r^	58
AZ70	MKP103; carbapenemase (KPC-3) deletion derivative of KPNIH1	Amp^r^	51
AZ71	hvKP1; hypervirulent K. pneumoniae K-type 2 isolate	Amp^r^	70
AZ94	Derivative of KPPR1 with apramycin resistance cassette at *att*::Tn*7* site	Apra^r^ Rif^r^	71
AZ63	AZ55 with pKD46 plasmid for lambda red recombination	Rif^r^ Str^r^ Spec^r^	This study
AZ101	Arrayed library; MKP103 with transposon element in *fimH*::*cam*	Cam^r^	51
AZ108	*fimH*::*cam* cassette from AZ101 into AZ63; plasmid cured	Rif^r^ Str^r^ Cam^r^	This study
AZ124	*ΔwcaJ* (CPS^−^) mutant of KPPR1S (AZ55)	Str^r^	35
AZ128	*wcaJ*^+^ (chromosomally complemented) derivative of AZ124	Str^r^	This study

a Amp^r^, ampicillin resistant; Apra^r^, apramycin resistant; Rif^r^, rifampin resistant; Str^r^, streptomycin resistant; Spec^r^, spectinomycin resistant; Cam^r^, chloramphenicol resistant.

The *wzi* gene codes for a conserved outer membrane protein involved in the attachment of capsular polysaccharide to the outer membrane. Sequence polymorphism in the *wzi* gene has been used to identify and characterize different isolates. K. pneumoniae AZ10 (*wzi* 372), an antibiotic-sensitive isolate, was made Str resistant as described previously, subsequently mouse GI passaged, and named AZ99 (52, 54). To construct the *fimH* mutant in the appropriate genetic background, PCR was carried out using Q5 polymerase (New England BioLabs [NEB]) with AZ101 genomic DNA as the template and primers *fimH upstream* (GGCGGTGATTAACGTCACCT) and *fimH downstream* (GATAGAGCAGCGTTTGCCAC), which give at least 500 bp of homology on either end of the transposon cassette. The PCR product was purified using the Qiagen MinElute kit. Lambda Red mutagenesis was carried out as described previously (55), and cells were recovered in super optimal broth with catabolite repression (SOC) media at 30°C with shaking overnight. Recovered bacteria were plated on selective LB agar containing chloramphenicol (50 μg/ml). Single colonies were purified and the mutation was confirmed by PCR. The complement of the Δ*wcaJ* strain was constructed by cloning flanking upstream and downstream regions of *wcaJ* into conjugation plasmid pKAS46 (56) using primers LT016-GATCTGCGCGCGATCGATATCCGCGGTGAAATGCATGTGTC and LT019-GCGCCAGCTGCAGGCGGCCGCGCAGAGAGTTCACGGTTACG and electroporating the plasmid into E. coli S17-1λpir (57). Conjugation and subsequent selection of complemented strain were carried out as described previously (58).

To determine *fim* promoter orientation, PCR was carried out using either *in vitro* samples from LB broth, single colonies from LB plates, or *in vivo* samples (fecal pellets) from mice infected with K. pneumoniae. Broth culture was spun down as described above and resuspended in an equal volume of distilled water (dH_2_O) and boiled for 5 min. Also, a single colony was resuspended in 10 μl of dH_2_O and boiled for 5 min. DNA was isolated from fecal pellets (100 mg) using Quick-DNA fecal/soil microbe microprep (Zymo Research). Five microliters of sample was used in PCR with OneTaq polymerase (NEB) with primers Cas168 (GGGACAGATACGCGTTTGAT) and Cas169 (GCCTAACTGAACGGTTTGA) as described previously (37). Purified PCR product was digested with restriction enzyme HinFI (NEB) for 1 h and resolved on a 1.2% agarose gel. As established previously, the “off orientation” of the *fim* promoter results in product bands of 496 bp and 321 bp, whereas the “on orientation” results in bands of 605 bp and 212 bp (37).

### Mouse infections for colonization and shedding.

Colonies of C57BL/6J (specific pathogen free [SPF]) mice obtained from Jackson Laboratory (Bar Harbor, ME) were bred and maintained in a standard animal facility at Biotech Place, Wake Forest Baptist Medical Center. All animal work was done according to the guidelines provided by the American Association for Laboratory Animal Science (AALAS) (59) and with the approval of the Wake Forest Baptist Medical Center Institutional Animal Care and Use Committee (IACUC). Five- to 7-week-old mice were infected and monitored through the course of the experiments. Food and water were removed from mice ∼4 h prior to inoculation. K. pneumoniae was grown overnight at 37°C with constant agitation, spun down in a centrifuge at 21,000 × *g* for 20 min, resuspended and washed once with PBS, and, finally, diluted in 2% sucrose-PBS. Mice were fed ∼10^6^ CFU/100 μl of K. pneumoniae in two 50-μl 2% sucrose-PBS doses an hour apart, from a pipette tip. Immediately afterwards, food was returned to mice. A similar protocol was followed for fasting mice and bacterial preparation for the gavage method of infection. Mice were treated by gavage with a 100-μl single dose of K. pneumoniae in 2% sucrose-PBS solution using a 20-gauge 1.5-in. single-use feeding needle. For both methods of infection, mouse scruffing without any anesthesia was used.

To quantify daily bacterial shedding, mice were removed from their housing and placed into isolation containers. Fecal pellets (∼0.02 g; approximately 2 pellets) were collected and placed into a 2-ml screw-cap tube (Fisherbrand; 02-682-558) along with at least 2 glass beads (BioSpec; 11079127). Samples were diluted 1:10 (weight/volume) in PBS. A Bead Mill 24 homogenizer (Fisherbrand) was used to homogenize the fecal pellets (2.1 power setting, 1 min). Afterwards, the tubes were spun in a minicentrifuge (Thermo Scientific; MySpin 6) to pellet out larger debris. Ten-fold serial dilutions were plated from the supernatant on appropriate antibiotic plates and incubated overnight at 30°C. Bacterial shedding was calculated in CFU per gram of feces. The limit of detection was 10^2^ CFU/g. Each mouse was uniquely marked so that the fecal shedding of each individual mouse could be tracked for the duration of the experiment.

To trigger an antibiotic-dependent K. pneumoniae supershedder phenotype, mice were infected orally with Str-resistant K. pneumoniae as described above. Four to 5 days postinoculation (p.i.), mice were treated by gavage with streptomycin (5 mg/200 μl) either once or on three consecutive days, and daily shedding was monitored post-antibiotic treatment. To determine the effect of neomycin treatment before K. pneumoniae infection, mice were treated by gavage with a single dose (5 mg/200 μl) 24 h before inoculation with MKP103, a derivative of the KPNIH1 isolate with a deletion of the KPC-3 carbapenemase-encoding gene (51). Bacterial counts were enumerated from fecal pellets as described above. To determine the role of continuous antibiotic treatment in the supershedder phenotype, the drinking water was replaced with water containing 1 g/liter of ampicillin 24 h before infection; mice were maintained on ampicillin-water for 10 day p.i., after which they were placed on regular water until the end of the experiment. K. pneumoniae fecal shedding was assessed up to 20 days postinfection and quantified as described above.

For competition experiments, mice were infected with a 1:1 mixture of AZ94 and the mutant of interest. Fecal shedding of both strains was assessed as described above. Fecal homogenates were plated on both apramycin (50 μg/ml) and Str (500 μg/ml) LB agar. The competitive index (CI) was calculated as described previously (60), using the following equation:

$${\text{log}}_{\text{1}0}\text{CI =}\frac{\text{ mutant output}/\text{WT output}}{\text{mutant input}/\text{WT input}}$$

A value of 0 would suggest that neither strain has an advantage. A value of >1 would suggest that the mutant has a competitive advantage, whereas a value of <1 would indicate that the WT has the advantage. Each CI experiment was carried out multiple times (biological replicate ≥ 2).

To determine colonization density in the GI tract, ileum, cecum, and colon were removed under sterile conditions immediately following CO_2_ (2 liters/min for 5 min) euthanasia of animals and subsequent cardiac puncture. The cecum, proximal colon, and a span of the terminal section of the ileum equal in length to the colon were removed from each animal. Organs were weighed and placed into individual 2-ml screw-cap tubes (Fisherbrand; 02-682-558) with at least 2 glass beads (BioSpec Products; 11079127). Samples were diluted 1:10 (weight/volume) in PBS and were homogenized and plated as described above. The limit of detection was 10^2^ CFU/g.

To determine colonization density in the kidneys, liver, and spleen, organs were removed under sterile conditions immediately following euthanasia as described above. Organs were weighed and placed in 15-ml conical tubes. For kidney and liver equal weight-to-volume PBS was added, and samples were homogenized using a PowerGen 700 homogenizer (power setting of 2 for 30 s), whereas for spleen, 10 times the volume of 1× PBS was added to the weight of the organ and homogenized as described above. The samples were plated as described above. The limit of detection for kidney and liver was 33 CFU/ml, and that for spleen was 10^2^ CFU/ml.

Oropharyngeal lavage was carried out with 200 μl of sterile PBS from a gavage needle inserted into the esophagus. The esophagus was exposed and cut transversely. A gavage needle, attached to a prefilled insulin syringe (BD) with 1× PBS, was then inserted into the cut esophagus, and PBS was collected from the mouth. The collected lavage was serially diluted and plated on appropriate antibiotic plates and incubated overnight at 30°C. The limit of detection for oral lavage was 33 CFU/ml.

### Transmission studies.

For 4:1 and 1:4 transmission experiments, C57BL/6J index mice (*n* = 1 or *n* = 4) at 5 to 7 weeks of age were infected with K. pneumoniae as described above, and feces were collected daily to determine colonization density of the GI tract. On day 4 p.i., contact mice (*n* = 4 or *n* = 1) were introduced to cages with the index mice. Feces of index and contact mice were collected and fecal shedding was quantified for at least 6 days postcohousing, for a total of 10 days for index mice and 6 days for contact mice. On day 10, the mice were euthanized as described above and the ileum, cecum, colon, and oral lavage samples of all mice were processed as described above to determine colonization density of K. pneumoniae.

In 1:4 transmission experiments, in which the administration of a single dose of antibiotic was assessed, index mice were infected with Str-resistant K. pneumoniae and fecal shedding of K. pneumoniae was quantified for 4 days p.i. On day 5 p.i., index mice were treated with streptomycin (5 mg/200 μl) via gavage and then cohoused with contact mice. Fecal shedding was determined daily for index and contact mice. In a continuous antibiotic challenge transmission study, an index mouse was put on water containing ampicillin (1 g/liter) 24 h before infection. The contact mice were placed on water containing ampicillin (1 g/liter) 24 h before introduction of the index mouse. Once the mice were cohoused, feces were collected daily from both index and contact mice and shedding was quantified to determine if any transmission events occurred.

To confirm that host-to-host transmission events occur through the fecal oral route, a metabolic cage (Tecniplast; catalog number 3700M022) was used. K. pneumoniae infections were carried out as described above. Four days postinfection, a contact mouse was introduced into the metabolic cage and feces were collected from both index and contact mice to determine transmission frequency. The metabolic cage’s metal wire floor allowed for fecal pellets and urine to fall through, separated through a special funnel and collected at the bottom. Therefore, the cage eliminated the ability of the mice to access fecal pellets for consumption (coprophagia). For each transmission study, ≥3 biologically independent experiments were carried out. Endpoint colonization of the lower GI tract (ileum, cecum, colon, and or feces) was considered a positive transmission event.

### Histology.

Mice were infected with either PBS (vehicle-only control) or the KPPR1S isolate. A subset of K. pneumoniae-infected mice were treated by gavage with streptomycin (single treatment; 5 mg/200 μl) to induce the supershedder state at 5 days p.i. As a positive control, mice were put on 3% (wt/vol) dextran sodium sulfate (DSS) (molecular weight, 50,000) in their drinking water *ad libitum* for 7 days. All the mice were euthanized at day 7 after initial treatment or infection. A total of 2.5 cm of colon immediately distal to the cecum was collected, washed with 1× PBS, and prepared using the Swiss roll method. Afterwards, the sample was preserved in 1:10 formalin (Fisherbrand; 305-510) and after 24 h was transferred to 70% ethanol. The samples were embedded in paraffin before being sectioned, mounted, and stained with hematoxylin and eosin (H&E). The resulting slides were scored by the Wake Forest Baptist Medical Center pathology department.

Liver was collected under sterile conditions from mock-infected mice and mice orally infected with hvKP1 and in extremis. Liver samples were cut in to sections about 6.5 mm and placed in 10% formalin (10 parts formalin to 1 part tissue). After 24 to 48 h, the samples were transferred to 70% ethanol and stored at 4°C until they were further processed for H&E and Gram staining, with scoring carried out as described above.

### Fecal microbiome analysis.

Fecal microbiome was examined according to previously described methods (61–63). Briefly, genomic DNA from 200 mg feces was extracted using the MoBio Powerfecal DNA kit (Qiagen, Valencia, CA) per the manufacturer’s instructions. Amplicon PCR of the V4 hypervariable region of the 16S rRNA gene was performed using the universal primers 515F (barcoded) and 806R according to the Earth Microbiome Project protocol (64). The amplicons were purified using AMPure magnetic beads (Agencourt), and the products were quantified with a Qubit-3 fluorimeter (Invitrogen). The final amplicon library was generated as previously described (64). An equimolar pooled library was sequenced on an Illumina MiSeq platform using a 2 × 300-bp reagent kit (MiSeq reagent kit v3; Illumina Inc.) for paired-end sequencing. The sequencing quality control was done with on-board MiSeq control software and MiSeq reporter (Illumina Inc.), and the obtained sequences were demultiplexed, quality filtered, clustered, and analyzed using QIIME software package (61, 62, 65, 66). Taxonomy classification was performed within QIIME based on 97% sequence similarity to the Greengenes database (65). Alpha-diversity and bacterial proportions were compared using Kruskal-Wallis test followed by pairwise Mann-Whitney test. Linear discriminatory analysis (LDA) effect size (LEfSe) was applied to identify discriminative features (unique bacterial taxa) that drive differences at different time points or in different groups (67). Hierarchical clustering and heat maps depicting the patterns of abundance were constructed within the R statistical software package (version 3.6.0; https://www.r-project.org/) using the heatmap.2 and ggplots packages.

### Statistical analysis.

All statistical analyses were performed using GraphPad Prism 8.0 (GraphPad Software, Inc., San Diego, CA). Unless otherwise specified, differences were determined using the Mann-Whitney U test (comparing two groups) or the Kruskal-Wallis test with Dunn’s postanalysis (comparing multiple groups).

## Supplementary Material

Supplemental file 1

Supplemental file 2
