# Supplementary material for: Animal Model To Study Klebsiella pneumoniae Gastrointestinal Colonization and Host-to-Host Transmission
Source: Infect Immun. 2020 Oct 19;88(11):e00071-20. doi: 10.1128/IAI.00071-20 (PMC7573435; doi:10.1128/IAI.00071-20)

Supplemental Figures.

Figure S1. Treatment of *K. pneumoniae* infected mice with PBS alone (200 µl) does not elicit a supershedder phenotype.

Figure S2. The colonization density in the GI tract (Ileum, cecum and colon) for the WT isolate, isogenic capsule deficient mutants (*ΔmanC* and *ΔwcaJ*) and the complemented strain $wcaJ^{+}$ was determined 15 days post-infection with median values shown. Limit of detection was 10^2 CFU/Gram of tissue. Differences in GI colonization determined using Kruskal-Wallis test. *, $p < 0.05$, **, $p < 0.01$, ***, $p < 0.001$, n.s, not significant.

Figure S3. (A.) Antibiotic treatment leads to development of supershedder phenotype in the index (Infected) mice and high transmission rates. Index mice were given ampicillin (1g/Liter) in drinking water a day before oral inoculation with *Kpn* isolate MKP103. Post-infection index mice were housed separately from the contact mice for 3 days. One day before the index mouse was moved in to the contact mice (n=4) cage they were also given ampicillin (1g/Liter) in drinking water. The contact mice quickly became colonized with MKP103 isolate, and both the index and contact mice displayed the supershedder phenotype (> 10^8 CFU/Gram of feces). Shown here are representation of two independent transmission studies. **(B.)** Fecal shedding data collected from mice given 10^6 CFU of KPPR1S housed in a metabolic cage to reduce coprophagia, and followed for up to 10 days post-infection. Also, shown is data from mice infected with KPPR1S (10^6 CFU) housed in regular cages for statistical comparison. Shown are median values, where each point represents a single mouse on a given day. Limit of detection (L.O.D) was 10^2 CFU/Gram of Feces. Mann-Whitney test used to determine the differences in daily fecal shedding. *, $p < 0.05$, n.s, not significant.

Figure S4. Fecal shedding differences between mice colonized with *Kpn* (KPPR1S) through oral feeding versus the oral gavage method. Mice were either fed or gavaged 10^6 CFU of *Kpn* in 100 µl of 2% sucrose-PBS. Shedding was collected daily up to 15 days post-infection. Mann-Whitney test used to determine the differences in daily fecal shedding. *, $p < 0.05$, **, $p < 0.01$.

Figure S5. Colonization by *Klebsiella pneumoniae* shows normal colonic mucosa. Displayed here are swiss rolls of colonic mucosa stained with hematoxylin and eosin staining. **(A.)** Mice given PBS alone and tissue prepared 7 days post-treatment (n=2). **(B.)** Colon tissue from mice prepared 7 days post 3% DSS daily treatment (n=2). Detailed here is the ulceration (long arrows) of the mucosa, with inflammation expanding the submucosa (*), and loss and fragmentation of individual muscle cells (short arrows). **(C.)** Mice infected with KPPR1S and tissue prepared 7 days post-infection (n=2). **(D.)** Mice infected with KPPR1S, given a single-dose of streptomycin (5 mg/ 200 µl) to trigger supershedder phenotype and tissue prepared two days post antibiotic treatment (n=2). Colonic epithelium of mice appears normal that are infected with KPPR1S and its supershedder phenotype, and is similar in morphology to the PBS control.

Table S1. List of bacterial species identified in the mice fecal pellets using 16s rRNA analysis

Supplemental Figure 1.

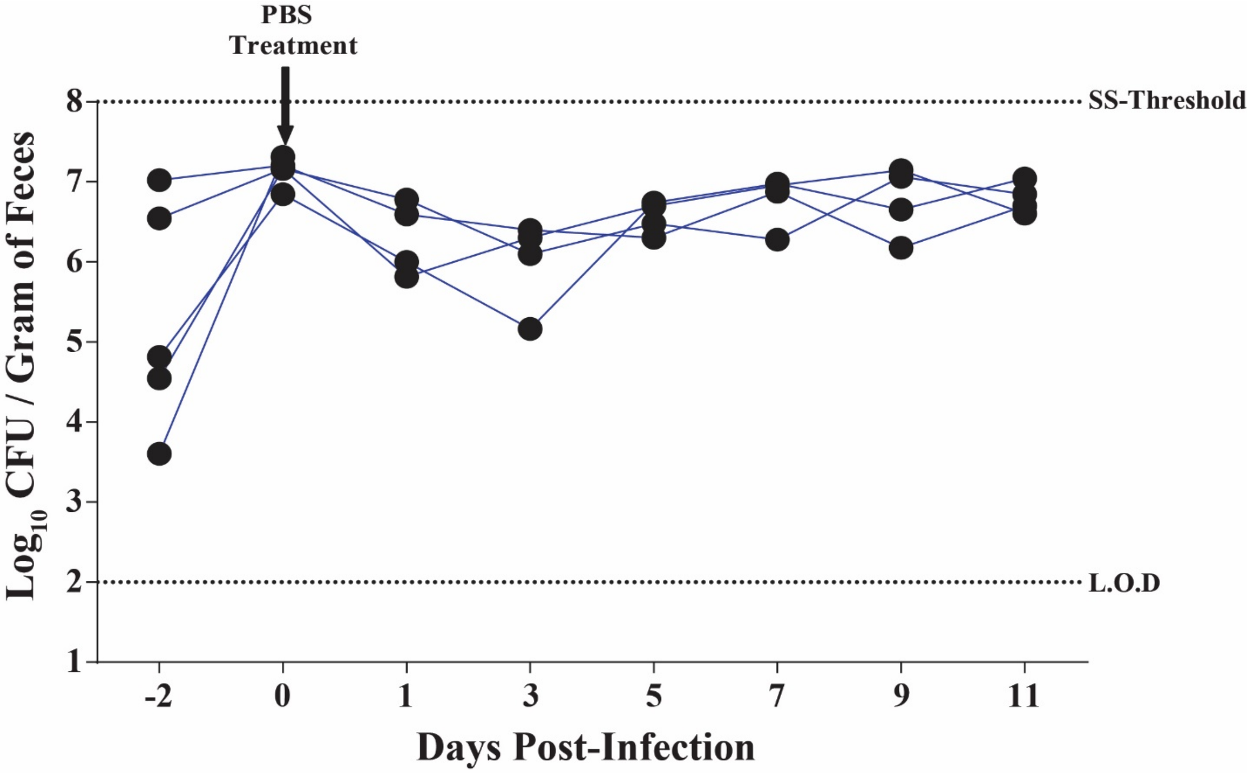

Supplemental Figure 2.

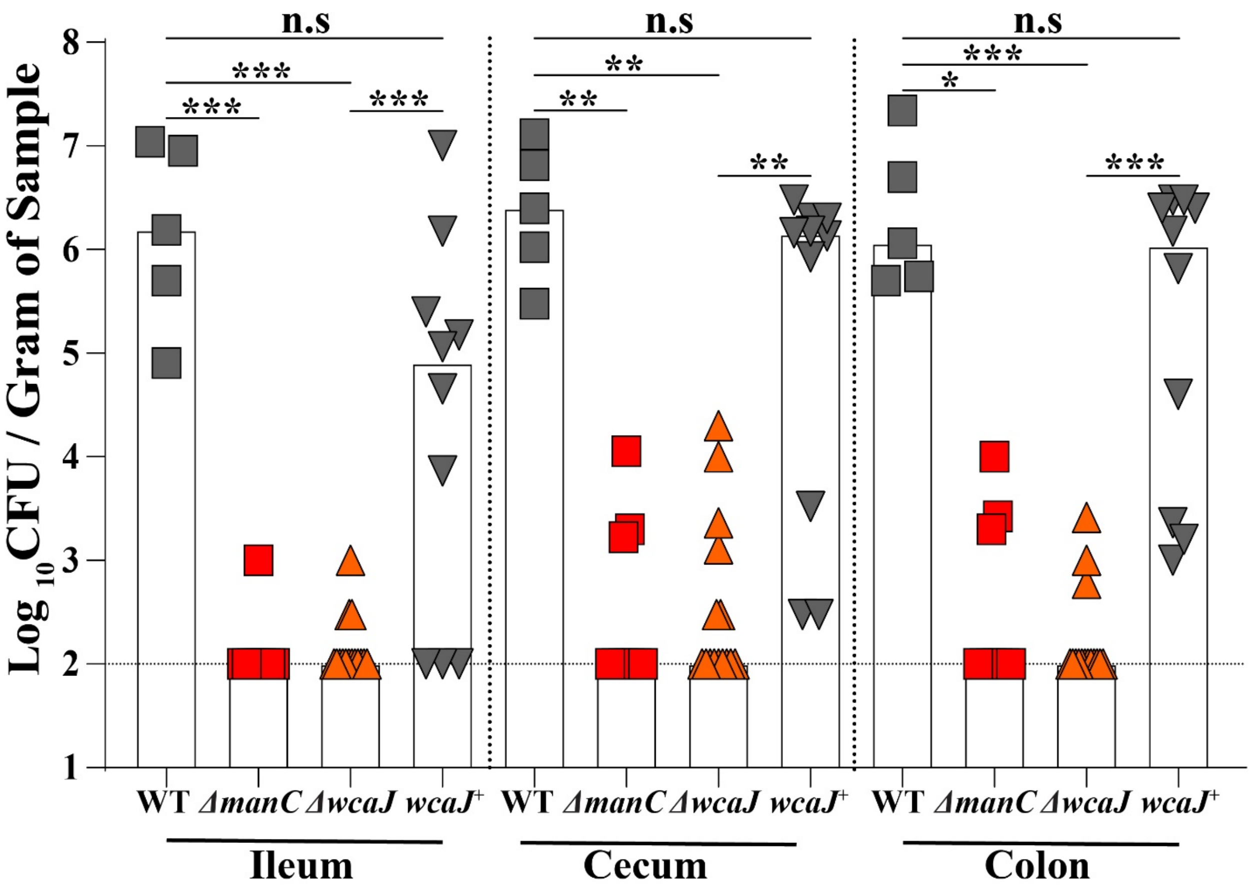

Supplemental Figure 3.

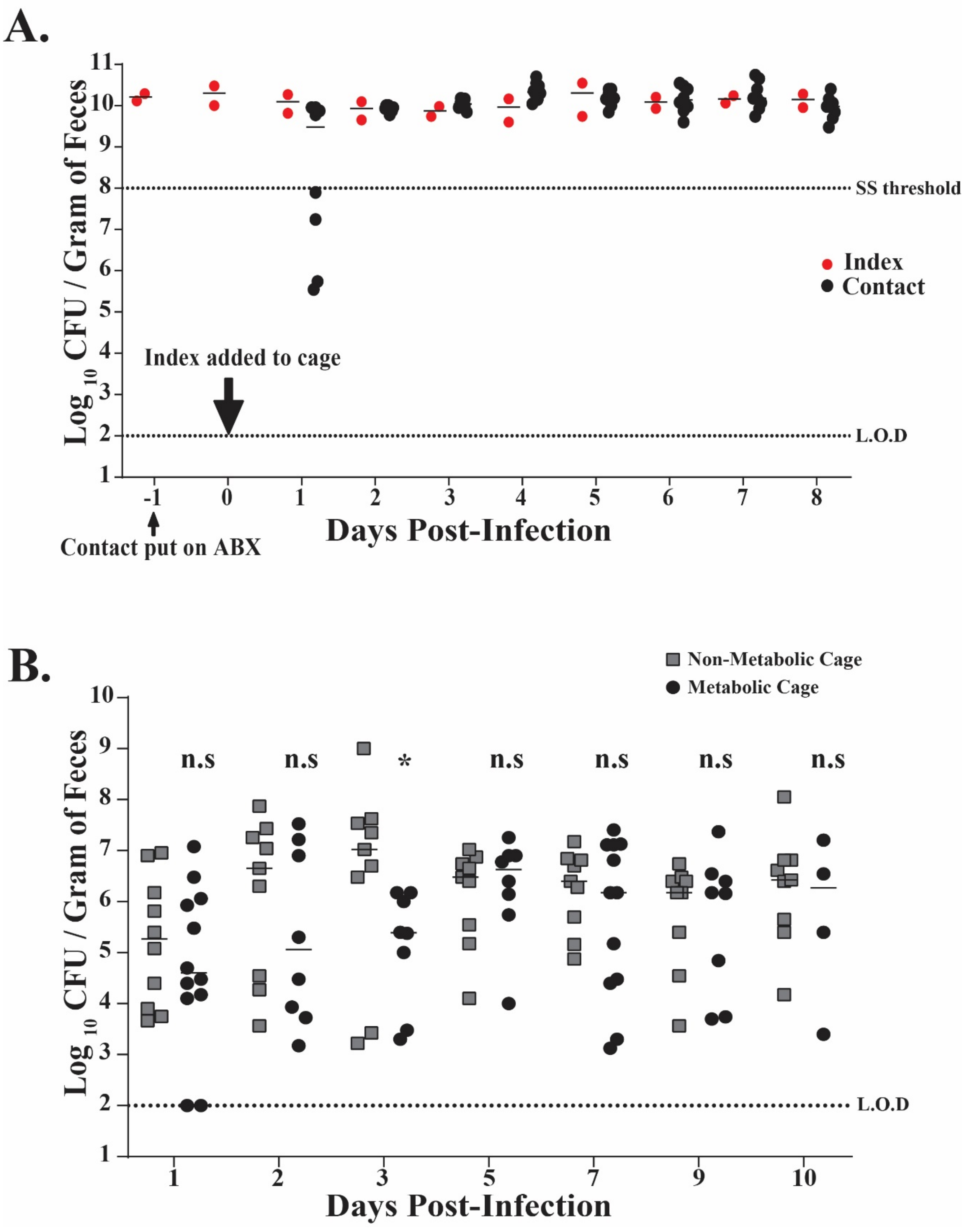

Supplemental Figure 4.

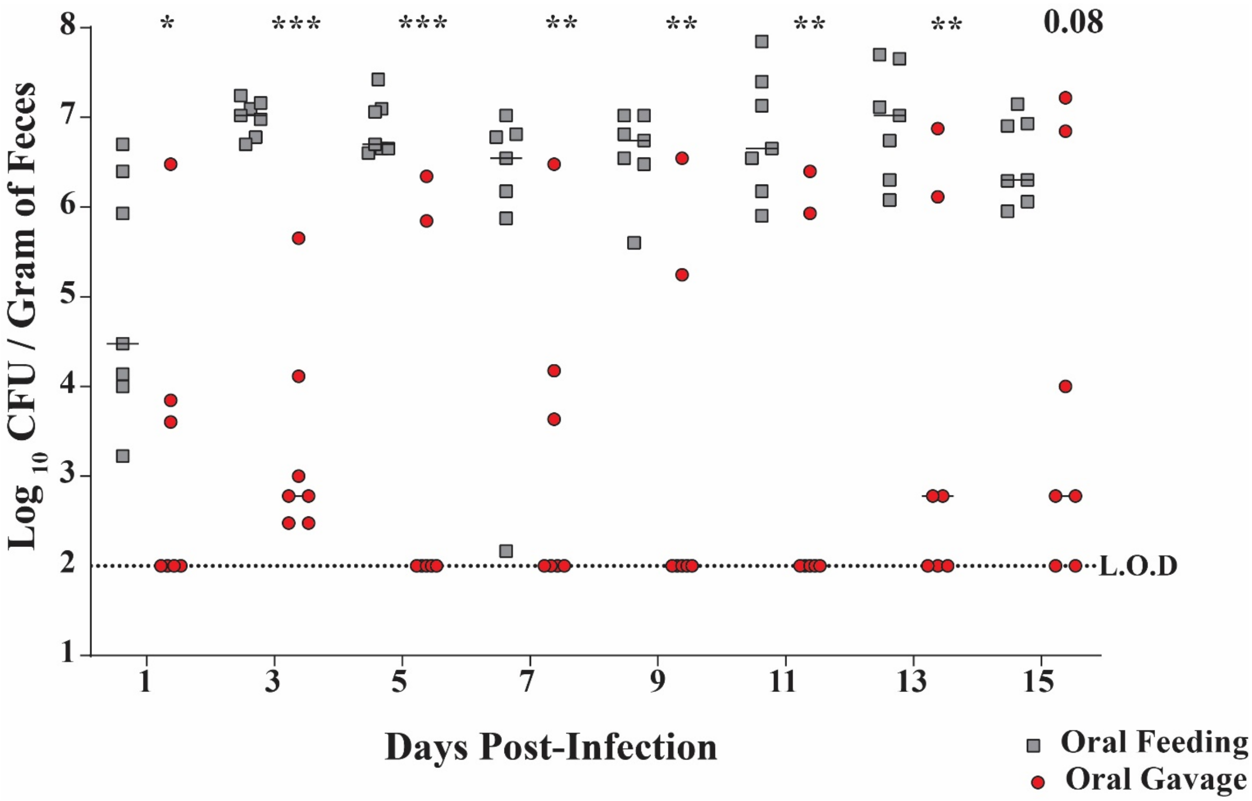

Supplemental Figure 5.

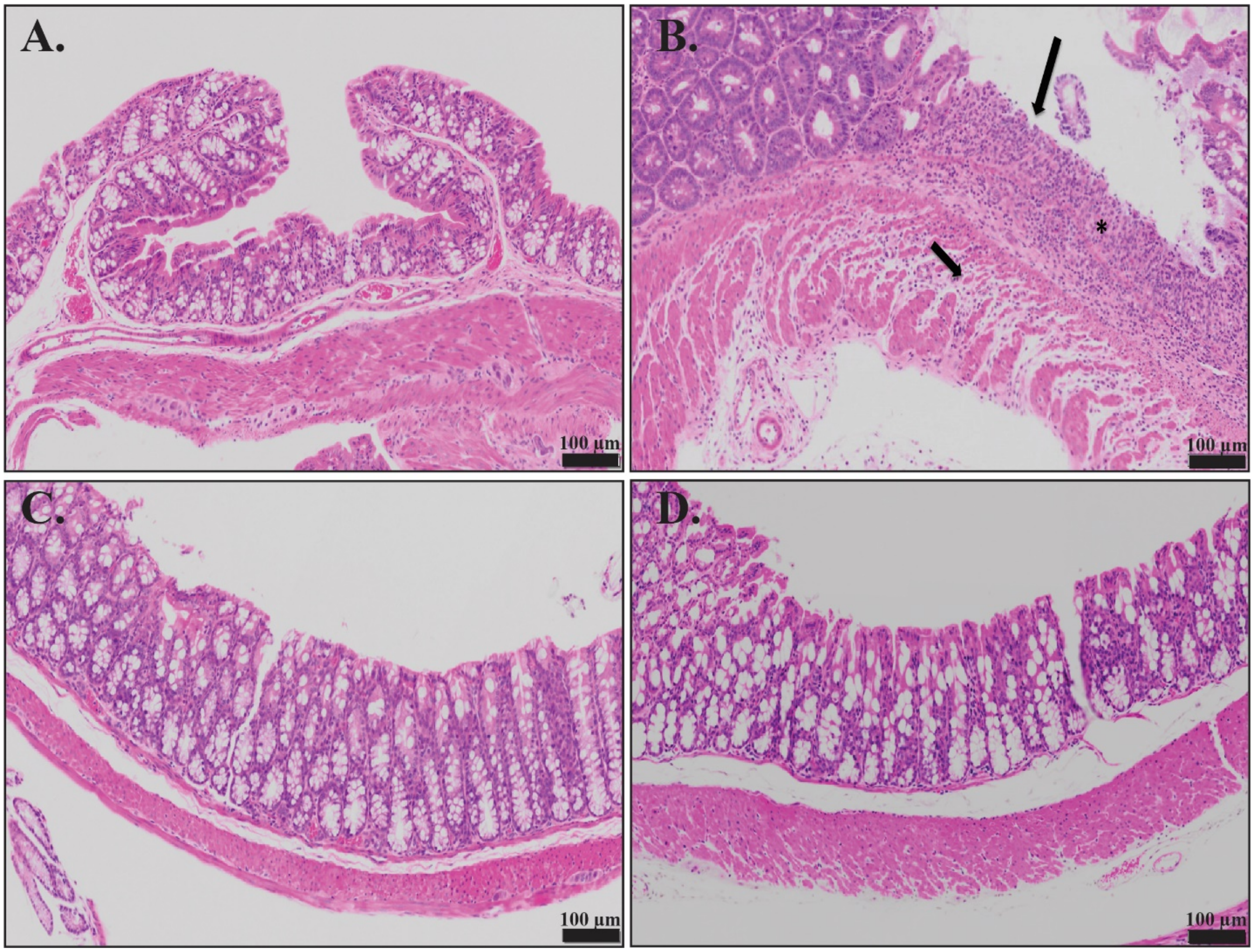

Supplement: Supplemental file 2 [file IAI.00071-20-s0002.pdf]
